# Natural Transformation of *Campylobacter jejuni* Occurs Beyond Limits of Growth

**DOI:** 10.1371/journal.pone.0045467

**Published:** 2012-09-26

**Authors:** Christina S. Vegge, Lone Brøndsted, Małgorzata Ligowska-Marzęta, Hanne Ingmer

**Affiliations:** Department of Veterinary Disease Biology, Faculty of Life Sciences, University of Copenhagen, Frederiksberg C, Denmark; Loyola University Medical Center, United States of America

## Abstract

*Campylobacter jejuni* is a human bacterial pathogen. While poultry is considered to be a major source of food borne campylobacteriosis, *C. jejuni* is frequently found in the external environment, and water is another well-known source of human infections. Natural transformation is considered to be one of the main mechanisms for mediating transfer of genetic material and evolution of the organism. Given the diverse habitats of *C. jejuni* we set out to examine how environmental conditions and physiological processes affect natural transformation of *C. jejuni*. We show that the efficiency of transformation is correlated to the growth conditions, but more importantly that transformation occurs at growth-restrictive conditions as well as in the late stationary phase; hence revealing that growth *per se* is not required for *C. jejuni* to be competent. Yet, natural transformation of *C. jejuni* is an energy dependent process, that occurs in the absence of transcription but requires an active translational machinery. Moreover, we show the ATP dependent ClpP protease to be important for transformation, which possibly could be associated with reduced protein glycosylation in the ClpP mutant. In contrast, competence of *C. jejuni* was neither found to be involved in DNA repair following DNA damage nor to provide a growth benefit. Kinetic studies revealed that several transformation events occur per cell cycle indicating that natural transformation of *C. jejuni* is a highly efficient process. Thus, our findings suggest that horizontal gene transfer by natural transformation takes place in various habitats occupied by *C. jejuni*.

## Introduction

The Gram-negative organism *Campylobacter jejuni* is the primary food borne bacterial pathogen in the developed world causing millions of gastroenteritis cases each year. Infections with *C. jejuni* typically results in acute illness with clinical symptoms ranging from mild to severe mucoid and bloody diarrhoea, and in rare cases the serious neurodegenerative Guillain Barré syndrome [1]. *C. jejuni* is a zoonotic pathogen and domestic poultry is an important reservoir, as consumption and handling of contaminated chicken meat products is one of the predominant routes of human infections [2]. *C. jejuni* is highly adapted to the avian gastrointestinal environment and it colonizes the gut of chickens to very high cell numbers without causing significant symptoms in the birds [3], [4]. *C. jejuni* is a fastidious organism, as it only grows under microaerobic conditions in a narrow temperature interval of approximately 30–46°C [5], [6]. In addition to a significant degree of host adaptation *C. jejuni* survives well in the external environment and contaminated water has been the cause of many cases of human gastroenteritis [7], [8], [9], [10].

The adaptive capacity of *C. jejuni* is in part attributed to a remarkable degree of genetic diversity [11], [12]. Studies have revealed substantial variation in genes encoding carbohydrate surface structures, flagella proteins and respiratory enzymes, which contribute to the virulence potential of *C. jejuni* upon infection but moreover also to survival in the external environment [13], [14], [15], [16], [17], [18]. The genetic diversity is caused by genomic rearrangements and hyper variable sequences but very importantly also by exchange of DNA between strains [19], [20], [21], [22]. *C. jejuni* is naturally competent for uptake of DNA from the environment and horizontal gene transfer has been shown to occur efficiently both *in vitro* and *in vivo* [21], [23].

Competence for natural transformation has been described for bacteria from a wide range of taxonomic groups [24], but the mechanisms of natural transformation have only been investigated in detail for a limited number of species. For most competent bacteria, exemplified by *Bacillus subtilis*, *Steptococcus pneumoniae* and *Haemophilus influenzae*, the development of competence is limited to a transient physiological state, tightly regulated by growth conditions and/or signal peptides [25], [26], [27], while other organisms, such as *Neisseria gonorrhoeae* and *Helicobacter pylori*, seem to be constitutively competent [28], [29].

Natural transformation by *C. jejuni* is far less characterized and improved knowledge of natural transformation of *C. jejuni* will therefore facilitate in understanding sources of strain diversity and moreover improve the tools for molecular investigations of this organism. It has been shown that the transformation frequency of *C. jejuni* is highly affected by the carbon dioxide concentration and to a minor extent by the growth phase [23], [30], while the levels of oxygen and temperature are reported to influence natural transformation of *C. coli* [31]. Several genes are required for natural transformation of *C. jejuni*, such as *Cj0011c*, *ctsD* (*Cj1474c*), *ctsE* (*Cj1471c*), *ctsF* (*Cj1470*), *ctsG* (*Cj1343c*), and *Cj1211*. They encode proteins with homology to a DNA receptor, type II secretions systems and/or pseudopilus proteins of other competent bacteria, respectively, and are therefore expected to be involved in uptake and transport of DNA in *C. jejuni* [32], [33], [34]. Likewise, the N-linked protein glycosylation (*pgl*) system is important for competence of *C. jejuni* [35].

To explore competence of *C. jejuni* in relation to environmental survival and adaptation, we have investigated the efficacy of natural transformation under conditions both restrictive and permissive for growth. Furthermore, we examined the significance of the Clp protease and central cellular processes on competence of *C. jejuni* to determine the boundaries of natural transformation. Our findings suggest that natural transformation occurs under a variety of environmental conditions and that the process may be important for the organism in multiple milieus.

## Results

### Natural Transformation of *C. jejuni* is Constitutive and Highly Efficient

For studying natural transformation of *C. jejuni* NCTC11168 we employed a previously described biphasic media system [23] composed of a solid agar layer and liquid medium layer with exponentially growing bacteria placed at 37°C under microaerobic conditions. As selective markers for DNA uptake and genomic integration, we routinely used genomic DNA carrying either an *rpsL^Sm^* mutation, providing resistance to streptomycin, or a Cam^R^ marker inserted into *tlp1,* a chromosomal locus not conferring growth defects *in vitro* [36].

The efficiency of natural transformation was evaluated both relative to the number of bacterial cells and the amount of added genomic DNA. When excess amounts of chromosomal Cam^R^ DNA was used in the transformation assay (CFU:DNA genome molecules = ca. 1∶10), 0.36% of the total CFUs obtained the resistance marker (Table 1). By limiting the amount of chromosomal DNA (CFU:DNA genome molecules = ca. 10∶1) the transformation efficiency could furthermore be evaluated relative to the amount of DNA, which showed 0.54% of the added DNA molecules to give rise to Cam^R^ transformants (Table 1). The number of Cam^R^ transformants represents cells having obtained a chromosomal region carrying the resistance marker, and these measured Cam^R^ transformants are only expected to constitute a minor fraction, while the actual number of transformed cells and the amount of DNA picked up from the environment very well could be substantially higher.

**Table 1:** Natural transformation efficiency of *C. jejuni* NCTC11168.

CFU:DNA	CFU/ml^a^	DNA^b^/ml	Transf./CFU^ac^	Transf./DNA molecule^ad^
ca. 1∶10^e^	3.0×10^8^±2.9×10^8^	6500 ng ≈ 3.5×10^9^ DNA molecules	3.6×10^−3^±1.8×10^−3^	−
ca. 10∶1^f^	3.6×10^8^±2.6×10^7^	65 ng ≈ 3.5×10^7^ DNA molecules	−	5.4×10^−3^±1.0×10^−3^

a Results are mean of three replicates with standard deviation.

b Chromosomal DNA from *C. jejuni* NCTC11168 Δ*tlp1::Cam^R^.*

c Limits of range tested: 10^−4^–10^0^ transformants/CFU.

d Limits of range tested: 10^−5^–10^−1^ transformants/DNA molecule.

e Excess amounts of DNA.

f Limited amounts of DNA.

The specificity of natural transformation by *C. jejuni* NCTC11168 was examined by transforming with DNA of different origin. *C. jejuni* NCTC11168 was readily transformed with chromosomal DNA from Strep^R^ mutants of two *C. jejuni* strains, DVI-SC11 and DVI-SC181 isolated from Danish chickens [37] (results not shown). In contrast, the addition of increasing amounts of DNA from the closely related species *Helicobacter pylori* and *Arcobacter butzleri* could not outcompete the transformation of isogenic DNA (Table S1). This is not a comprehensive experiment, but these data suggest natural transformation of *C. jejuni* NCTC11168 to be species- but not strain-specific.

**Figure 1:**
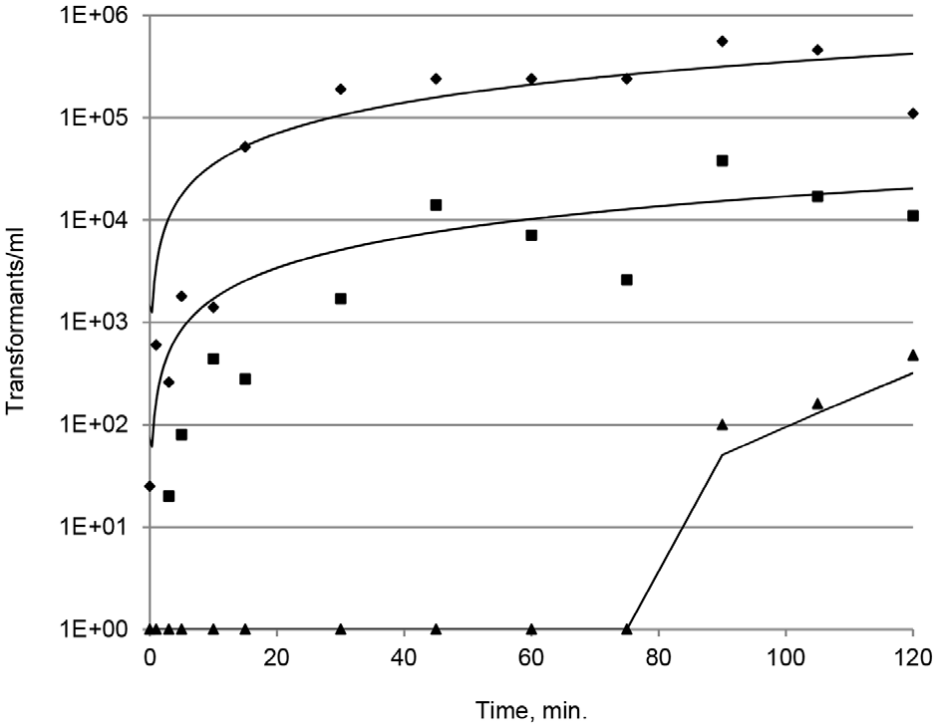
Kinetics of natural transformation. *C. jejuni* transformed (t = 0) with equal amounts of isogenic chromosomal DNA carrying a Strep^R^ or a Cam^R^ resistance marker, respectively. At various time points (0–120 min.) following addition of DNA the transformation was terminated by adding DNase I. Single and double transformants were scored by plating on selective agar. Results are representative from three independent experiments. Diamonds, Cam^R^ transformants, Squares, Strep^R^ transformants; Triangles, Cam^R^ Strep^R^ double transformants.

To examine the kinetics of natural transformation in more detail, we investigated if several transformation events occur per cell cycle by transforming simultaneously with chromosomal DNA encoding either Cam^R^ or Strep^R^ resistance. Aliquots of an exponentially growing *C. jejuni* NCTC11168 culture were distributed to 13 samples and each sample equally transformed with 2 µg/ml of both Cam^R^ DNA and Strep^R^ DNA. Individual transformations were stopped at various time points (0–120 min.) by the addition of DNase I, and following 2 h of phenotypic expression single and double transformants were scored by plating on selective agar. Cam^R^ or Strep^R^ transformants was obtained already after a few minutes of exposure to DNA and a saturated level of transformants, i.e. a stable number of resistant colonies, was obtained after approximately 30 min of co-cultivation with DNA (Figure 1), indicating that single DNA uptake had occurred. The time of transformation saturation was the same for both resistance markers although differences were observed in the number of transformants/ml. The generation time of *C. jejuni* NCTC11168 is approximately 2 h under the experimental conditions (results not shown), and thus the saturation level of single transformants was obtained already at a quarter of a generation. The appearance of transformants carrying both resistance markers, i.e. bacterial cells have takes up and integrated multiple DNA fragments, occurred after approximately 1.5 h, which is less than a generation, and the fraction of these double transformants increased steadily up to the 2 h time limit of the experiment (Figure 1). These results show that DNA uptake and natural transformation of *C. jejuni* is a continuous and fast process and that multiple transformation events can occur within a cell cycle.

### Quantification of Extracellular DNA in *C. jejuni* NCTC11168 Cultures

In order to determine the concentration of extracellular DNA in *C. jejuni* cultures, a fluorescent nucleic stain was applied to quantitatively assess the amount of double stranded DNA in the culture supernatant of *C. jejuni* NCTC11168 Δ*tlp1*. Thus, we found approximately 0.04 µg/ml DNA to be present in the supernatants of early stationary cultures (OD_600_ 1.0 ≅ ca. 2.5×10^9^ CFU/ml) in MEMα minimal media supplemented with 20 mM sodium pyruvate and 40 µM FeSO_4_. The result represents the difference between treated and untreated DNase I samples and is corrected for background fluorescence from the media and DNase I. The measured extracellular DNA concentration was furthermore confirmed in natural transformation assays using the culture supernatants as DNA source (results not shown). The measured extracellular DNA concentration corresponds to approximately one molecule of a *C. jejuni* genome per 100 CFU under the investigated conditions.

### *C. jejuni* is Competent Under Growth-restrictive Conditions

We observed *C. jejuni* NCTC11168 to be competent in both exponential and late stationary growth phase, although the number of transformants per CFU was approximately 400 times lower for bacterial cells in late stationary phase compared to cells in the exponential growth phase (Table 2). This observation prompted us to investigate if *C. jejuni* is competent under conditions not supporting growth. Growth of *C. jejuni* is restricted to conditions mimicking the environment of the gastrointestinal tract, i.e. microaerobic conditions and temperatures between 30–46°C [5], [6]. Natural transformations were carried out and compared for various conditions inside and outside this window of growth (Table 3). The level of natural transformation was highest under microaerobic conditions at 42°C, which reflects the conditions of the avian gut. However, transformation was found to occur at all tested conditions although at highly variable degree. Generally, the temperature had a much greater influence on the transformation than the concentration of oxygen, since decreased temperatures caused a more severe reduction of the transformation than did changing the oxygen concentration from microaerobic to aerobic conditions. Noteworthy, the level of natural transformation of *C. jejuni* was almost equal at aerobic and microaerobic conditions at 20, 30 and 37°C, respectively. On the other hand, an anaerobic environment was significantly less supportive of natural transformation as compared to microaerobic conditions (Table 3).

**Table 2:** Natural transformation in exponential and stationary growth phases.

Growthphase	OD600	Aggregates^a^	CFU/ml^b^	Transformants/CFU^b^
Exponential	0.05	No	4.3×10^9^±7.4×10^8^	2.3×10^−3^±3.2×10^−4^
Latestationary	2.2	Yes	4.3×10^8^±7.6×10^7^	5.9×10^−6^±2.6×10^−6^

a Aggregates of bacterial cells resuspended before transformation.

b Mean of four replicates with standard deviations.

**Table 3:** Natural transformation of *C. jejuni* at varying temperature and atmospheric conditionsa,b.

Temperature	Oxygen level		
	Aerobic	Microaerobic	Anaerobic
20°C	0.11±0.13	0.16±0.27	Nd.^c^
30°C	15.1±8.92	19.6±7.37	Nd.^c^
37°C	97.3±10.3	Index 100^b^ ±55.5	28.1±12.9
42°C	130±50.8	353±37.8	16.2±6.49

a Exponentially growing cells were supplied with isogenic chromosomal DNA at 1∶2.5 ratio (CFU:DNA) and transformation was allowed to proceed for 4 h before plating on selective agar. Results are the average of three independent experiments with two replicates.

b Results are normalized to the number of transformants per ml at 37°C under microaerophilic conditions.

c Not determined.

**Figure 2:**
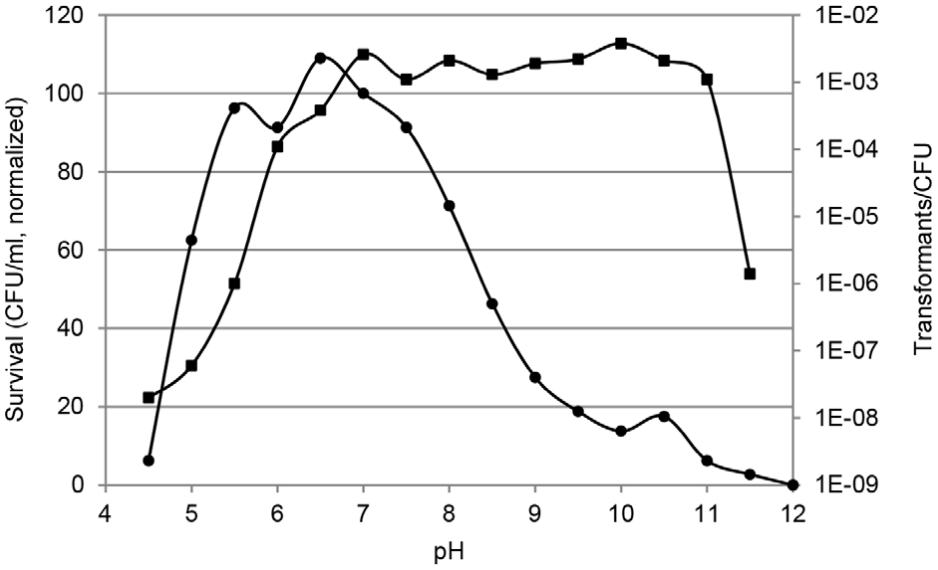
Natural transformation at variable pH. Natural transformation of *C. jejuni* in BHI broth with pH values from 4 to 12. Values of CFU/ml are normalized to the value at pH 7, while transformants/CFU are exact values. Results are representative from three independent experiments. Squares, transformants/CFU. Circles, survival, CFU/ml normalized to pH 7.

To further explore the effect of growth restrictive conditions we investigated natural transformation and survival of *C. jejuni* at variable pH (Figure 2). From pH 5 to ca. 8.5 more than 50% of bacterial cells survived within the 4 h experiment, while the fraction of viable cells rapidly dropped outside this pH interval. In contrast, natural transformation occurred very efficiently in the pH interval 7 to 11 with a relatively constant level of viable CFU’s obtaining the resistance marker, while at pH conditions outside this interval the number of transformants per CFU was reduced by 10–10^5^ fold (Figure 2). From these results it is clear that the pH interval for natural transformation is different from the pH interval supporting growth, since higher pH correlates with lower survival but higher number of transformants.

Taken together these results show that natural transformation of *C. jejuni* can take place under several conditions not supporting growth. Moreover, we found no indications that stress enhances or induces competence of *C. jejuni*, since the levels of transformation were found to peak at optimal growth conditions.

### Competence is Dependent on the Level of Cellular Energy and Protein Synthesis

In order to explore the regulation of natural transformation, we investigated the impact of cell density and the contribution of central cellular processes, such as respiration, translation and transcription to transformation.

First, we observed the transformation frequency of *C. jejuni* to increase at reduced cell density. The cell densities of exponentially growing cultures (OD600 0.3–0.4) were reduced 20 fold by dilution with growth media and transformed with *rpsL^Sm^* DNA in the standard assay. This caused a 50 fold increase in the number of transformants per CFU for the diluted cultures compared to the original undiluted cultures (Diluted cultures: 4.1×10^−5^±5.7×10^−7^ transf./CFU and undiluted cultures: 8.2×10^−7^±2.1×10^−7^ transf./CFU. The results are mean of two independent experiments).

*C. jejuni* perform oxidative phosphorylation to obtain ATP, and an active electron transport chain is therefore crucial for energy generation. The cellular energy level of *C. jejuni* can be reduced by respiratory inhibitors that inhibit the transfer of electrons in central enzymes of the electron transport chain [36], [38]. When we examined natural transformation in the presence of the respiratory inhibitors HQNO and sodium azide we found that the transformation mechanism of *C. jejuni* is an energy requiring process strongly depending on an active electron transport chain, as transformation was strongly inhibited by these compounds (Table 4). In addition, the energy level prior to natural transformation is important for transformation, since the number of transformats per CFU was also greatly reduced by the presence of the respiratory inhibitors just before the addition of DNA (Table 4).

**Table 4:** Natural transformation is dependent on active electron transport.

Respiratory inhibitor	Time of addition	CFU/ml^c^	Transformants/CFU^c^
Negative control	–	Index 100	Index 100
HQNO^d^	During transformation^b^	88	0.71
HQNO^d^	Prior to transformation^a^	57	25
Sodium azide^e^	During transformation^b^	65	0.61
Sodium azide^e^	Prior to transformation^a^	64	4.5

a Exponentially growing cells were incubated with the respiratory inhibitor for 2 h prior to a wash out of compound and addition of DNA.

b The respiratory inhibitor were added to the exponentially growing cells together with the DNA.

c Normalized to the negative control.

d 100 µg/ml.

e 500 µM.

**Figure 3:**
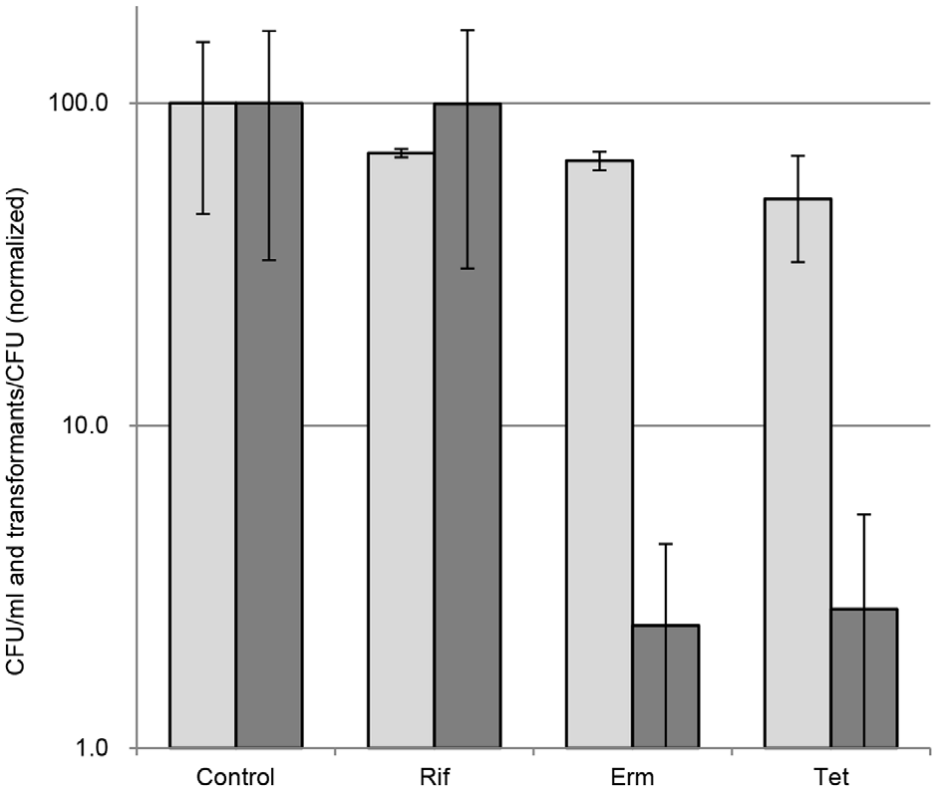
The importance of transcription and translation for competence. Natural transformation in the presence of transcriptional or translational inhibitors. CFU/ml (light grey) and transformants/CFU (dark grey). The results represent a mean of three replicates with standard deviations. Data are normalized to the value of the negative control (no additive). Rif, 500 µg/ml rifampicin; Erm, 2 µg/ml erythromycin; Tet, 20 µg/ml tetracycline. White bars, CFU/ml; grey bars, transformants/CFU.

To determine if the level of gene and protein expression *per se* affects competence of *C. jejuni*, natural transformation was investigated while inhibiting the transcriptional and translational processes, respectively. We found natural transformation of *C. jejuni* was inhibited by the translation inhibitors erythromycin and tetracycline, while largely being unaffected by the transcriptional inhibitor, rifampicin (Figure 3). *C. jejuni* is intrinsically resistant to rifampicin, but the resistance can be overcome by increasing the concentration of rifampicin [39]. The inhibitory effect on transformation by the translation inhibitors was not caused by lethal effects of these antibiotics, since the reduction in viability was less than 50% and therefore much less than the reduction in transformation frequency (Figure 3). Natural transformation of *C. jejuni* thus seems to be modulated at the level of protein synthesis.

### Natural Transformation of *C. jejuni* is Dependent on the ClpP Protease

**Figure 4:**
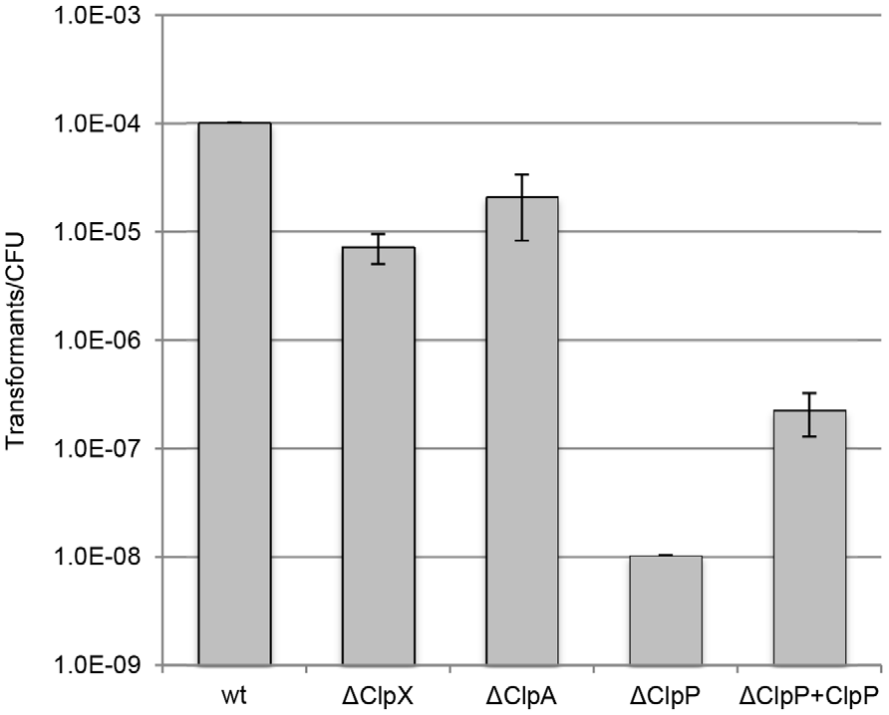
Natural transformation of Clp mutants. Natural transformation efficiency of *C. jejuni* Clp deletion mutants and the complemented ClpP mutant (ΔClpP+ClpP) transformed with excess amounts of isogenic chromosomal Strep^R^ DNA. The results represent a mean of three replicates with standard deviations.

The Clp proteolytic complex is involved in regulation of competence in *B. subtilis* and *S. pneumonia* and to investigate if this complex also is involved competence of *C. jejuni*, mutants of the ClpP protease and the ClpA and ClpX ATPases were analyzed in the standard transformation assay. This showed the Δ*clpP* mutant of *C. jejuni* to be practically untransformable, while mutants lacking either the ClpX or the ClpA ATPases showed approximately 93 and 80% reduced transformation, respectively, when compared to the wild-type (wt) strain (Figure 4). Competence of the Δ*clpP* strain could be restored by inserting a copy of the *clpP* gene into a nonessential chromosomal locus of the mutant (resulting in strain Δ*clpP*+*clpP*), although the obtained level of transformation was lower than that of the wt strain (Figure 4). These experiments show that competence of *C. jejuni* is dependent on ClpP.

### Natural Transformation of *C. jejuni* is Neither Involved in DNA Repair Nor Provides a Growth Benefit

*C. jejuni* lacks a typical SOS response and many of the DNA repair genes [20], and therefore we speculated that this organism may use external DNA to repair damaged DNA. However, the efficiency of transformation was unaltered following exposure to DNA damaging agents such as UV light or Mitomycin C (Table S2). Also, survival following UV light exposure did not increase when the bacteria were allowed to recover in the presence of excess amounts of DNA (Table S2). Furthermore, external DNA did not significantly alter the frequency of spontaneous nalidixic acid resistant colonies within three generations after the addition of DNA (results not shown). These data suggest that natural transformation does not serve to repair DNA following DNA damage in *C. jejuni*.

To investigate if *C. jejuni* utilizes DNA as a nutritional source, we compared the growth of the wt strain in minimal media (MEMα without nucleosides) in the presence and absence of excess amounts of extracellular DNA. However, the addition of 10 µg/ml chromosomal DNA did not result in higher cell numbers than the cultures without added DNA (Figure S1). Likewise, co-cultivation of a competent strain (*rpsL*) with a competent-deficient strain (Δ*clpP*) in minimal media with and without excess amounts of extracellular DNA did not reveal any growth or survival advantages for the competent strain from early exponential to late stationary phase (results not shown).

## Discussion

In this study, we have explored natural transformation of *C. jejuni* in order to determine how the transformation process is affected by environmental conditions and to assess the physiological impact of this process. We find that natural transformation in *C. jejuni* occurs with a high efficiency (Table 1), and that the uptake of DNA from the extracellular environment must be a continuous and fast process resulting in multiple transformation events per cell cycle (Figure 1).

Horizontal gene transfer occurs actively when strains of *C. jejuni* are co-cultivated either *in vitro* or *in vivo* [21]. This gene transfer is prevented by DNase and culture supernatants may serve as DNA source for transformation thus indicating that the exchange of genetic material is independent of cell-to-cell contact (results not shown) [32]. Therefore, genomic DNA of *C. jejuni* must be present in the extracellular environment either via release or by cell lysis. We estimated the extracellular DNA concentration of *C. jejuni* NCTC11168 to be ca. 0.04 µg/ml, which corresponds to approximately one *C. jejuni* genome per 100 CFU under the investigated conditions. This is less than what have been described for *N. gonorrhoeae*, which actively releases DNA via a type IV secretion system [40], [41], but comparable to what have been observed for *N. meningitides* where DNA is released in a process relying on autolysins [42]. However, such autolysins have not been described for *C. jejuni*.

*C. jejuni* was most efficiently transformed at optimal growth conditions, i.e. in the exponential growth phase, at 37–42°C in a microaerobic atmosphere and pH ca. 7 (Table 2–3, Figure 2), which is consistent with previous observations from *C. jejuni* and *C. coli* [23], [30], [31]. The level of transformation was significantly reduced by inhibition of the electron transport chain by respiratory inhibitors (Table 4), while the addition of fresh nutrients by culture dilution had a positive effect on the transformation frequency. Taken together these results indicate the efficiency of natural transformation by *C. jejuni* is connected to growth rate and thus may be correlated with the physiological fitness of the organism.

Competence development of *B. subtilis, H. influenzae* and *S. pneumoniae* is induced upon nutrient limitation, pheromones and/or by environmental stress [25], [26], [27], [43]. In contrast, we find *C. jejuni* to be competent in both the exponential and late stationary growth phase as well as at growth limiting conditions such as low temperature, at aerobic and anaerobic levels of oxygen and in alkaline environments, that are conditions restrictive for growth but not for physiological activity of *C. jejuni* [5]. In this aspect, *C. jejuni* resembles *N. gonorrhoeae* in being competent during all phases of growth [44]. However, natural transformation of *N. gonorrhoeae* is dependent on the expression of essential pili proteins and specific DNA uptake sequences [45], which so far have not been identified in *C. jejuni*. Based on our findings we consider natural transformation of *C. jejuni* to be a constitutive process that operates regardless of environmental conditions and beyond the conditional boundaries supporting growth.

In agreement with the notion that *C. jejuni* is constitutively competent we found no evidence of natural transformation being directly dependent on cell density. Again, this is in contrast to the regulation of competence in *B. subtilis* and *S. pneumoniae* in which extracellular competence-stimulating peptides initiate the transcription of competence genes in the stationary and exponential growth states, respectively [43]. Central regulators of competence in these organisms are the key transcriptional regulator ComK and the alternative sigma factor ComX, respectively, as well as the ClpP protease, which degrades ComK and ComX [43], [46], [47]. Neither ComK nor ComX homologues are found in *C. jejuni*, but we found an interesting common characteristic of competence between *C. jejuni* and these organisms, since the ClpP protease strongly affect natural transformation of *C. jejuni* (Figure 4). Likewise, *C. jejuni* mutants of the complementary ClpX and ClpA ATPases showed decreased ability to be naturally transformed (Figure 4) suggesting that each of the two Clp ATPases could function together with ClpP in this process. A polar effect on the downstream deformylase (*def*) gene has previously been reported for the *clpP* mutant [48]. However, competence of the *clpP* mutant could partly be restored by reintroducing the wt *clpP* allele, and we therefore conclude that the competence deficiency of the mutant was indeed attributed to the ClpP protease. The function of the Clp proteolytic complex in competence of *C. jejuni*, however, remains unknown, and can be either directly by processing of competence proteins or indirectly by processing of other proteins with downstream effects on natural transformation. A previous proteomic analysis showed the Δ*clpP* mutant to carry altered levels of several proteins with as various functions such as motility and glycosylation [48]. Protein glyscosylation is important for competence of *C. jejuni* [35], and since the Δ*clpP* mutant was shown to contain reduced amounts of the UDP-GlcNac/Glc-4-epimerase (gne/galE) [48], which is a central enzyme of the glycosylation pathway [49], and a *gne* mutant furthermore displays reduced level of natural transformation [50], we hypothesize that the competence deficiency of the Δ*clpP* mutant is, at least partly, caused by reduced protein glycosylation. However, it is likely that other factors are also involved in the lacking competence of the Δ*clpP* mutant.

Natural transformation in *C. jejuni* is dependent on protein synthesis, since inhibition of translation caused a severe reduction in the transformation efficiency, while transcriptional inhibition did not reveal the same effect (Figure 3). This finding points to a translational control of competence in *C. jejuni*, which can be caused either by a requirement of *de novo* synthesis of essential competence proteins or physical instability/rapid turnover of such proteins. This consideration is in agreement with observations of reduced transformation frequency of *H. pylori* following gentamicin treatment [51], and a correlation between trans-translational tagging and competence of *H. pylori* [52]. Moreover, natural transformation of *N. gonorrhoeae* requires active protein synthesis in addition to a type IV pilus assembly apparatus [53], [54].

DNA damaging agents cause an induction of essential competence genes in *H. pylori* and *S. pneumoniae*, and the level of natural transformation increase following DNA damage in these organisms [51], [55]. In this study, it was explored if a similar correlation between DNA damage and natural transformation exists in *C. jejuni*. However, we found the level of natural transformation to be unaffected by DNA damage caused by Mitomycin C and UV light and moreover, high concentrations of available DNA in the extracellular environment did not increase either the recovery from DNA damage by UV light or the spontaneous mutation rate (Table S2 and results not shown). Likewise, *E. coli* has been shown to utilize DNA as a nutritional source [56], [57], but a similar benefit of the constitutive competence of *C. jejuni* could not be identified (Figure S1).

In conclusion, this study shows that competence of *C. jejuni* is a constitutive process dependent on protein synthesis and the ClpP protease. The efficiency of natural transformation seems correlated to growth, but nevertheless *C. jejuni* is able to take up DNA even under conditions not supportive of proliferation. We therefore hypothesize that horizontal gene transfer by natural transformation is important for milieu adaptation of *C. jejuni* both inside the gastrointestinal tract and in the external environment.

## Experimental Procedures

### Cultivation of *C. jejuni*

The original clinical isolate of *C. jejuni* NCTC11168 (11168-O) [58] was routinely cultivated on Blood agar Base II (Oxoid) supplemented with 5% bovine blood or in Brain Heart Infusion broth (BHI) (Oxoid). The bacteria were incubated at 37°C in a controlled atmosphere (6% O_2_, 6% CO_2_, 4% H_2_, and 84% N_2_) in a MACS VA1000 microaerophil workstation (Don Witley Scientific) unless otherwise stated. When cultivated in broth, serial dilutions of bacteria in 8–12 ml media were made in 90 mm Petri dishes and incubated overnight as described above. Preceding all experiments, *C. jejuni* cultures were investigated for contamination by phase contrast microscopy and cultures of the appropriate growth phase selected by optical density measurements.

### Natural Transformation Assays

For a standard natural transformation assay, 200 µl of an exponentially growing *C. jejuni* in BHI (optical density 600 nm: 0.1–0.3) was transferred to a 2.0 ml cryo tube containing 1 ml BHI-agar. This biphasic culture was preincubated for 2 h under microaerobic conditions at 37°C followed by the addition of 10–400 ng DNA and transformation for 2–4 h under the same conditions. The transformed culture was subsequently diluted in isotonic sodium chloride solution and CFUs were scored by plating on Blood agar Base II, while transformants, which had obtained resistance marker(s), were scored by plating on Blood agar Base II supplemented with 20 µg/ml chloramphenicol, 50 µg/ml streptomycin or 30 µg/ml kanamycin when appropriate. Control experiments confirmed that the transformation process was terminated by plating on selective media. Chromosomal DNA purified by the DNeasy Blood & Tissue Kit (Qiagen) from *C. jejuni* NCTC11168 Δ*tlp1::Cam^R^* or *C. jejuni* NCTC11168 *rpsL^Sm^* [36], [59] were routinely used for natural transformation assays. For test of DNA specificity, chromosomal DNA from *C. jejuni* DVI-SC11 Strep^R^ or *C. jejuni* DVI-SC181 Strep^R^ [37] were applied. For competition experiments with foreign DNA, increasing amounts (0–200 ng) of chromosomal DNA from *Helicbacter pylori* ATCC700392 or *Arcobacter butzleri* ATCC4916 were supplied together with 20 ng of isogenic chromosomal DNA. For investigations on cell density and transformation efficiency, exponentially growing bacteria were diluted 20 fold in BHI prior to transformation with the standard transformation assay.

### Kinetics of Natural Transformation

300 µl aliquots of an exponentially growing culture of *C. jejuni* NCTC11168 were dispensed to 13 tubes and each sample transformed with 2 µg/ml isogenic chromosomal DNA carrying a Strep^R^ or a Cam^R^ resistance marker, respectively. The transformations were terminated at various time points (0–120 min.) by the addition of 0.017 units of DNase I (Fermentas), and following 2 h of phenotypic expression, single and double transformants were scored by plating on selective agar.

### Quantification of Extracellular DNA

*C. jejuni* NCTC11168 Δ*tlp1* was cultivated to early stationary phase in MEMα (Life Technologies) supplemented with 20 mM sodium pyruvate and 40 µM FeSO_4_. Bacterial cells were removed by centrifugation and the concentration of doublestanded DNA in the sterile filtered supernatants were determined by fluorescence spectrometry and the Quant-iT™ Picogreen® dsDNA assay kit (Life Technologies) using the manufacturer’s instructions. Background fluorescence levels were disregarded by determining the difference between untreated and DNase I treated samples and correcting for fluorescent from the media and DNase I. The extracellular DNA concentration was furthermore confirmed by natural transformation assays of *C. jejuni* wt with the *C. jejuni* Δ*tlp1* supernatants in comparison with a standard row of transforming DNA. An estimate on the number of extracellular DNA genomes per CFU was made using an estimated molecular weight of the *C. jejuni* NCTC11168 genome (1.6×10^6^ bp * 660 g/(mole*bp)), the Avogadro constant (6×10^23^ molecules/mole), the measured DNA concentration and CFU/ml.

### Natural Transformation Under Stress Conditions

Natural transformations under environmental stress conditions was determined with the standard natural transformation assay described above with the following exceptions: The effect of temperature and oxygen was analyzed by adding 400 ng of DNA to transform *C. jejuni* NCTC11168 wt for four hours at 20, 30, 37 or 42°C under aerobic conditions, or in sealed containers with microaerobic or anaerobic environments provided by CampyGen or AnaeroGen sachets (Oxoid), respectively. The effect of pH was analyzed by harvesting by centrifugation exponentially growing *C. jejuni* NCTC11168 bacteria, resuspending in sodium chloride solution, and transferring to BHI broth with pH values from 4–12. Biphasic media was not applied for these samples. Following two hours of preincubation, 50 ng of isogenic Cam^R^ chromosomal DNA was added and the transformation allowed to proceed for two hours before plating on selective and non-selective solid media.

### Inhibition of Electron Transport Chain, Transcription and Translation

Various compounds were added to the standard natural transformation assay carried out without biphasic media. The respiration was inhibited by the addition of 100 µg/ml HQNO (2-n-Heptyl-4-hydroxyquinoline N-oxide) (Alexis) or 500 µM sodium azide (Fluka) either only during the preincubation step or during the transformation step. For inhibition of transcription and translation, the preinkubation step was omitted and 500 µg/ml rifampicin, 2 µg/ml erythromycin, or 20 µg/ml tetracycline, respectively, were added together with the DNA. Following two hours of transformation the inhibitory compounds were washed away by exchange of media, the transformation processes terminated by addition of 0.015 units DNase I (Fermentas) and phenotypic expression allowed to proceed for two hours before plating on selective and non-selective solid media.

### Construction of Complemented ClpP Mutant and Natural Transformation of Clp Mutants

Deletion mutants of *C. jejuni* NCTC11168 *clpP*, *clpA* and *clpX* have been described previously [48]. For complementation of the Δ*clpP* mutant we applied a pKfdxA vector [60] kindly donated by Dr Duncan Gaskin. This vector contains a BsmBI restriction site located downstream from fdxA promoter. A divergent promoter controls expression from a gene conferring Kanamycin resistance. Both promoters are located between flanking sequences of pseudogene *cj0046* that allow for recombination of any gene cloned into BsmBI site. The *clpP* gene was amplified by with primers clpP-for (5′ ATCGTCTCACATGTTTATTCCTTATGTT) and clpP-rev (5′ ATCGTCTCACATGTCATTTAAAACTTTT) containing BsmBI restriction sites. The resulting PCR product and the pKfdxA vector were then cut with the Esp3I enzyme (an isoschizomer of BsmBI, Fermentas) and ligated, placing the gene under the control of the fdxA promoter. Ligated constructs were sequenced to confirm the presence of the insert, its correct orientation, as well as lack of point mutations. Vectors with successfully cloned *clpP* gene were then introduced by electroporation into strain *C. jejuni clpP::cat* [48] and the transformants were selected for Chloramphenicol and Kanamycin resistance. Natural transformation of *C. jejuni* NCTC11168 Δ*clpP*, Δ*clpA*, Δ*clpX* and Δ*clpP+clpP* was investigated in the standard natural transformation assays using 400 ng of isogenic chromosomal Strep^R^ DNA.

### DNA Damage, Mutation Rate and Natural Transformation

Natural transformation of *C. jejuni* wt was monitored during DNA damage caused by 10–500 ng/ml Mitomycin C (Sigma-Aldrich) in standard transformation assays without preincubation and biphasic media. Natural transformation and recovery following DNA damage caused by UV irradiation was examined as follows: Exponentially growing *C. jejuni* NCTC1168 bacteria were harvested by centrifugation, resuspended in isotonic salt solution to OD_600_ ca. 0.3 and treated with 0–80 J/m^2^ UV in an ultraviolet crosslinker (UVP). Following UV exposure, 2×100 µl aliquots of bacterial suspension from each UV treatment were added either 100 µl BHI +400 ng isogenic Cam^R^ chromosomal DNA or just 100 µl BHI. Following 2 h recovery at 37°C in a microaerobic environment, samples were added 3 µl DNase I (Fermentas) and plated on non-selective and selective agar for scoring of transformants and CFU/ml. Transformants/CFU were compared between UV treated and untreated samples, while CFU/ml were compared for samples recovered with or without added DNA. The spontaneous mutation rate during natural transformation of *C. jejuni* was analyzed by plating and enumerating Nal^R^ colonies on Blood agar Base II supplemented with 50 µg/ml nalidixic acid at various time points following natural transformation with excess amounts of isogenic strep^R^ chromosomal DNA.

### Competence and Long-term Growth Competition with Excess Amounts of DNA

Bacteria of an exponentially growing culture of *C. jejuni* NCTC1168 in BHI were harvested by centrifugation, washed in minimal media and resuspended to OD_600_ 0.03 in minimal media MEMα without nucleosides (Life Technologies, catalog no 32561) supplemented with 40 µM FeSO_4_. The culture was subsequently divided in two, and one fraction supplemented with 10 µg/ml of isogenic chromosomal Cam^R^ DNA. CFU/ml was determined at the start of the experiment and after 24 h of incubation at 37°C under microaerobic conditions. Growth competition experiments with *C. jejuni* NCTC1168 *rpsL^Sm^* and Δ*clpP::Kan^R^* [48] were carried out at 37°C under microaerobic conditions in the defined minimal medium MEMα (Life Technologies) supplemented with 40 µM FeSO_4_ and 10 µg/ml chromosomal DNA isolated from *C. jejuni* Δ*tlp1.* Cultures were inoculated with approximately10^4^ CFU/ml of each strain and subpopulation titers were determined periodically for four days by plating on selective media. The experiments were performed in duplicates.

## Supporting Information

Figure S1 **Growth of** ***C. jejuni*** **NCTC11168 is not supported by extracellular DNA.** Growth of *C. jejuni* NCTC11168 in minimal media (MEMα medium without nucleotides catalog no 32561, Life Technologies) without (white) and with 10 µg/ml of isogenic chromosomal DNA (grey). The results represent a mean of four replicates with standard deviations.
(TIF)

Table S1 **DNA from** ***A. butzleri*** **and** ***H. pylori*** **cannot outcompete natural transformation of isogenic DNA.**
(PDF)

Table S2 **Natural transformation and survival of** ***C. jejuni*** **following DNA damage.**
(PDF)

## References

[1] Wassenaar TM, Blaser MJ (1999) Pathophysiology of *Campylobacter jejuni* infections of humans. Microbes Infect 1: 1023–1033. 10617934 10.1016/s1286-4579(99)80520-6

[2] Lee MD, Newell DG (2006) *Campylobacter* in poultry: filling an ecological niche. Avian Dis 50: 1–9. 16617973 10.1637/7474-111605R.1

[3] Jacobs-Reitsma W, Lyhs U, Wagenaar J (2008) *Campylobacter* in the food supply. In: Nachamkin I, C. M Szymanski, and M. J Blaser editor. *Campylobacter*. 3 ed. Washington, DC: ASM Press. 627–644.

[4] Shane SM, Stern NJ (2003) *Campylobacter* infection. In: Saif YM, editor. Diseases of Poultry. Ames, Iowa: Iowa State Press. 615–630.

[5] Hazeleger WC, Wouters JA, Rombouts FM, Abee T (1998) Physiological activity of *Campylobacter jejuni* far below the minimal growth temperature. Appl Environ Microbiol 64: 3917–3922. 9758819 10.1128/aem.64.10.3917-3922.1998 PMC106578

[6] Bolton FJ, Coates D (1983) A study of the oxygen and carbon dioxide requirements of thermophilic *Campylobacters* . J Clin Pathol 36: 829–834. 6408142 10.1136/jcp.36.7.829 PMC498399

[7] Richardson G, Thomas DR, Smith RM, Nehaul L, Ribeiro CD, et al (2007) A community outbreak of *Campylobacter jejuni* infection from a chlorinated public water supply. Epidemiol Infect 135: 1151–1158. 17288640 10.1017/S0950268807007960 PMC2870681

[8] Engberg J, Gerner-Smidt P, Scheutz F, Moller Nielsen E, On SL, et al (1998) Water-borne *Campylobacter jejuni* infection in a Danish town–-a 6-week continuous source outbreak. Clin Microbiol Infect 4: 648–656. 11864264 10.1111/j.1469-0691.1998.tb00348.x

[9] Karagiannis I, Sideroglou T, Gkolfinopoulou K, Tsouri A, Lampousaki D, et al (2010) A waterborne *Campylobacter jejuni* outbreak on a Greek island. Epidemiol Infect 138: 1726–1734. 20836911 10.1017/S0950268810002116

[10] Gubbels SM, Kuhn KG, Larsson JT, Adelhardt M, Engberg J, et al. (2012) A waterborne outbreak with a single clone of *Campylobacter jejuni* in the Danish town of Køge in May 2010. Scand J Infect Dis. 10.3109/00365548.2012.655773 22385125

[11] Dorrell N, Mangan JA, Laing KG, Hinds J, Linton D, et al (2001) Whole genome comparison of *Campylobacter jejuni* human isolates using a low-cost microarray reveals extensive genetic diversity. Genome Res 11: 1706–1715. 11591647 10.1101/gr.185801 PMC311159

[12] Pearson BM, Pin C, Wright J, I’Anson K, Humphrey T, et al (2003) Comparative genome analysis of *Campylobacter jejuni* using whole genome DNA microarrays. FEBS Lett 554: 224–230. 14596944 10.1016/s0014-5793(03)01164-5

[13] Duong T, Konkel ME (2009) Comparative studies of *Campylobacter jejuni* genomic diversity reveal the importance of core and dispensable genes in the biology of this enigmatic food-borne pathogen. Curr Opin Biotechnol 20: 158–165. 19346123 10.1016/j.copbio.2009.03.004 PMC2769087

[14] Hofreuter D, Tsai J, Watson RO, Novik V, Altman B, et al (2006) Unique features of a highly pathogenic *Campylobacter jejuni* strain. Infect Immun 74: 4694–4707. 16861657 10.1128/IAI.00210-06 PMC1539605

[15] Phongsisay V, Perera VN, Fry BN (2006) Exchange of lipooligosaccharide synthesis genes creates potential Guillain-Barre syndrome-inducible strains of *Campylobacter jejuni* . Infect Immun 74: 1368–1372. 16428786 10.1128/IAI.74.2.1368-1372.2006 PMC1360302

[16] Leonard EE 2nd, Takata T, Blaser MJ, Falkow S, Tompkins LS, et al (2003) Use of an open-reading frame-specific *Campylobacter jejuni* DNA microarray as a new genotyping tool for studying epidemiologically related isolates. J Infect Dis 187: 691–694. 12599089 10.1086/368268

[17] Poly F, Threadgill D, Stintzi A (2004) Identification of *Campylobacter jejuni* ATCC 43431-specific genes by whole microbial genome comparisons. J Bacteriol 186: 4781–4795. 15231810 10.1128/JB.186.14.4781-4795.2004 PMC438563

[18] Gilbert M, Godschalk PC, Karwaski MF, Ang CW, van Belkum A, et al (2004) Evidence for acquisition of the lipooligosaccharide biosynthesis locus in *Campylobacter jejuni* GB11, a strain isolated from a patient with Guillain-Barre syndrome, by horizontal exchange. Infect Immun 72: 1162–1165. 14742567 10.1128/IAI.72.2.1162-1165.2004 PMC321600

[19] Nuijten PJ, van den Berg AJ, Formentini I, van der Zeijst BA, Jacobs AA (2000) DNA rearrangements in the flagellin locus of an *flaA* mutant of *Campylobacter jejuni* during colonization of chicken ceca. Infect Immun 68: 7137–7140. 11083841 10.1128/iai.68.12.7137-7140.2000 PMC97826

[20] Parkhill J, Wren BW, Mungall K, Ketley JM, Churcher C, et al (2000) The genome sequence of the food-borne pathogen *Campylobacter jejuni* reveals hypervariable sequences. Nature 403: 665–668. 10688204 10.1038/35001088

[21] de Boer P, Wagenaar JA, Achterberg RP, van Putten JP, Schouls LM, et al (2002) Generation of *Campylobacter jejuni* genetic diversity *in vivo* . Mol Microbiol 44: 351–359. 11972775 10.1046/j.1365-2958.2002.02930.x

[22] Karlyshev AV, Champion OL, Churcher C, Brisson JR, Jarrell HC, et al (2005) Analysis of *Campylobacter jejuni* capsular loci reveals multiple mechanisms for the generation of structural diversity and the ability to form complex heptoses. Mol Microbiol 55: 90–103. 15612919 10.1111/j.1365-2958.2004.04374.x

[23] Wang Y, Taylor DE (1990) Natural transformation in *Campylobacter* species. J Bacteriol 172: 949–955. 2404960 10.1128/jb.172.2.949-955.1990 PMC208523

[24] Johnsborg O, Eldholm V, Havarstein LS (2007) Natural genetic transformation: prevalence, mechanisms and function. Res Microbiol 158: 767–778. 17997281 10.1016/j.resmic.2007.09.004

[25] Hamoen LW, Venema G, Kuipers OP (2003) Controlling competence in *Bacillus subtilis*: shared use of regulators. Microbiology 149: 9–17. 12576575 10.1099/mic.0.26003-0

[26] Claverys JP, Havarstein LS (2002) Extracellular-peptide control of competence for genetic transformation in *Streptococcus pneumoniae* . Front Biosci 7: d1798–1814. 12133809 10.2741/claverys

[27] VanWagoner TM, Whitby PW, Morton DJ, Seale TW, Stull TL (2004) Characterization of three new competence-regulated operons in *Haemophilus influenzae* . J Bacteriol 186: 6409–6421. 15375121 10.1128/JB.186.19.6409-6421.2004 PMC516621

[28] Stingl K, Muller S, Scheidgen-Kleyboldt G, Clausen M, Maier B (2010) Composite system mediates two-step DNA uptake into *Helicobacter pylori* . Proc Natl Acad Sci U S A 107: 1184–1189. 20080542 10.1073/pnas.0909955107 PMC2824268

[29] Hamilton HL, Dillard JP (2006) Natural transformation of *Neisseria gonorrhoeae*: from DNA donation to homologous recombination. Mol Microbiol 59: 376–385. 16390436 10.1111/j.1365-2958.2005.04964.x

[30] Wilson DL, Bell JA, Young VB, Wilder SR, Mansfield LS, et al (2003) Variation of the natural transformation frequency of *Campylobacter jejuni* in liquid shake culture. Microbiology 149: 3603–3615. 14663092 10.1099/mic.0.26531-0

[31] Kim JS, Kim JW, Kathariou S (2008) Differential effects of temperature on natural transformation to erythromycin and nalidixic acid resistance in *Campylobacter coli* . Appl Environ Microbiol 74: 6121–6125. 18708520 10.1128/AEM.01075-08 PMC2565985

[32] Jeon B, Muraoka W, Sahin O, Zhang Q (2008) Role of *Cj1211* in natural transformation and transfer of antibiotic resistance determinants in *Campylobacter jejuni* . Antimicrob Agents Chemother 52: 2699–2708. 18505858 10.1128/AAC.01607-07 PMC2493120

[33] Jeon B, Zhang Q (2007) Cj0011c, a periplasmic single- and double-stranded DNA-binding protein, contributes to natural transformation in *Campylobacter jejuni* . J Bacteriol 189: 7399–7407. 17693521 10.1128/JB.01012-07 PMC2168429

[34] Wiesner RS, Hendrixson DR, DiRita VJ (2003) Natural transformation of *Campylobacter jejuni* requires components of a type II secretion system. J Bacteriol 185: 5408–5418. 12949093 10.1128/JB.185.18.5408-5418.2003 PMC193740

[35] Larsen JC, Szymanski C, Guerry P (2004) N-linked protein glycosylation is required for full competence in *Campylobacter jejuni* 81–176. J Bacteriol 186: 6508–6514. 15375132 10.1128/JB.186.19.6508-6514.2004 PMC516609

[36] Vegge CS, Brøndsted L, Li YP, Bang DD, Ingmer H (2009) Energy taxis drives *Campylobacter jejuni* toward the most favorable conditions for growth. Appl Environ Microbiol 75: 5308–5314. 19542337 10.1128/AEM.00287-09 PMC2725471

[37] Knudsen KN, Bang DD, Andresen LO, Madsen M (2006) *Campylobacter jejuni* strains of human and chicken origin are invasive in chickens after oral challenge. Avian Dis 50: 10–14. 16617974 10.1637/7376-051005R.1

[38] Myers JD, Kelly DJ (2005) A sulphite respiration system in the chemoheterotrophic human pathogen *Campylobacter jejuni* . Microbiology 151: 233–242. 15632441 10.1099/mic.0.27573-0

[39] Lin J, Michel LO, Zhang Q (2002) CmeABC functions as a multidrug efflux system in *Campylobacter jejuni* . Antimicrob Agents Chemother 46: 2124–2131. 12069964 10.1128/AAC.46.7.2124-2131.2002 PMC127319

[40] Dillard JP, Seifert HS (2001) A variable genetic island specific for *Neisseria gonorrhoeae* is involved in providing DNA for natural transformation and is found more often in disseminated infection isolates. Mol Microbiol 41: 263–277. 11454218 10.1046/j.1365-2958.2001.02520.x

[41] Hamilton HL, Dominguez NM, Schwartz KJ, Hackett KT, Dillard JP (2005) *Neisseria gonorrhoeae* secretes chromosomal DNA via a novel type IV secretion system. Mol Microbiol 55: 1704–1721. 15752195 10.1111/j.1365-2958.2005.04521.x

[42] Lappann M, Claus H, van Alen T, Harmsen M, Elias J, et al (2010) A dual role of extracellular DNA during biofilm formation of *Neisseria meningitidis* . Mol Microbiol 75: 1355–1371. 20180907 10.1111/j.1365-2958.2010.07054.x

[43] Claverys JP, Prudhomme M, Martin B (2006) Induction of competence regulons as a general response to stress in gram-positive bacteria. Annu Rev Microbiol 60: 451–475. 16771651 10.1146/annurev.micro.60.080805.142139

[44] Biswas GD, Sox T, Blackman E, Sparling PF (1977) Factors affecting genetic transformation of *Neisseria gonorrhoeae* . J Bacteriol 129: 983–992. 14116 10.1128/jb.129.2.983-992.1977 PMC235038

[45] Chen I, Dubnau D (2004) DNA uptake during bacterial transformation. Nat Rev Microbiol 2: 241–249. 15083159 10.1038/nrmicro844

[46] Turgay K, Hahn J, Burghoorn J, Dubnau D (1998) Competence in *Bacillus subtilis* is controlled by regulated proteolysis of a transcription factor. EMBO J 17: 6730–6738. 9890793 10.1093/emboj/17.22.6730 PMC1171018

[47] Robertson GT, Ng WL, Foley J, Gilmour R, Winkler ME (2002) Global transcriptional analysis of *clpP* mutations of type 2 *Streptococcus pneumoniae* and their effects on physiology and virulence. J Bacteriol 184: 3508–3520. 12057945 10.1128/JB.184.13.3508-3520.2002 PMC135132

[48] Cohn MT, Ingmer H, Mulholland F, Jørgensen K, Wells JM, et al (2007) Contribution of conserved ATP-dependent proteases of *Campylobacter jejuni* to stress tolerance and virulence. Appl Environ Microbiol 73: 7803–7813. 17933920 10.1128/AEM.00698-07 PMC2168155

[49] Bernatchez S, Szymanski CM, Ishiyama N, Li J, Jarrell HC, et al (2005) A single bifunctional UDP-GlcNAc/Glc 4-epimerase supports the synthesis of three cell surface glycoconjugates in *Campylobacter jejuni* . J Biol Chem 280: 4792–4802. 15509570 10.1074/jbc.M407767200

[50] Fry BN, Feng S, Chen YY, Newell DG, Coloe PJ, et al (2000) The *galE* gene of *Campylobacter jejuni* is involved in lipopolysaccharide synthesis and virulence. Infect Immun 68: 2594–2601. 10768949 10.1128/iai.68.5.2594-2601.2000 PMC97464

[51] Dorer MS, Fero J, Salama NR (2010) DNA damage triggers genetic exchange in *Helicobacter pylori* . PLoS Pathog 6: e1001026. 20686662 10.1371/journal.ppat.1001026 PMC2912397

[52] Thibonnier M, Thiberge JM, De Reuse H (2008) Trans-translation in *Helicobacter pylori*: essentiality of ribosome rescue and requirement of protein tagging for stress resistance and competence. PLoS One 3: e3810. 19043582 10.1371/journal.pone.0003810 PMC2584231

[53] Merz AJ, So M, Sheetz MP (2000) Pilus retraction powers bacterial twitching motility. Nature 407: 98–102. 10993081 10.1038/35024105

[54] Long CD, Tobiason DM, Lazio MP, Kline KA, Seifert HS (2003) Low-level pilin expression allows for substantial DNA transformation competence in *Neisseria gonorrhoeae* . Infect Immun 71: 6279–6291. 14573647 10.1128/IAI.71.11.6279-6291.2003 PMC219589

[55] Prudhomme M, Attaiech L, Sanchez G, Martin B, Claverys JP (2006) Antibiotic stress induces genetic transformability in the human pathogen *Streptococcus pneumoniae* . Science 313: 89–92. 16825569 10.1126/science.1127912

[56] Finkel SE, Kolter R (2001) DNA as a nutrient: novel role for bacterial competence gene homologs. J Bacteriol 183: 6288–6293. 11591672 10.1128/JB.183.21.6288-6293.2001 PMC100116

[57] Palchevskiy V, Finkel SE (2006) *Escherichia coli* competence gene homologs are essential for competitive fitness and the use of DNA as a nutrient. J Bacteriol 188: 3902–3910. 16707682 10.1128/JB.01974-05 PMC1482900

[58] Gaynor EC, Cawthraw S, Manning G, MacKichan JK, Falkow S, et al (2004) The genome-sequenced variant of *Campylobacter jejuni* NCTC 11168 and the original clonal clinical isolate differ markedly in colonization, gene expression, and virulence-associated phenotypes. J Bacteriol 186: 503–517. 14702320 10.1128/JB.186.2.503-517.2004 PMC305761

[59] Bæk KT, Vegge CS, Skorko-Glonek J, Brøndsted L (2011) Different contributions of HtrA protease and chaperone activities to *Campylobacter jejuni* stress tolerance and physiology. Appl Environ Microbiol 77: 57–66. 21075890 10.1128/AEM.01603-10 PMC3019702

[60] Shaw FL, Mulholland F, Le Gall G, Porcelli I, Hart DJ, et al. (2012) Selenium-dependent biogenesis of formate dehydrogenase in *Campylobacter jejuni* is controlled by the fdhTU accessory genes. J Bacteriol. 10.1128/JB.06586-11 PMC3416564 22609917

