# Supplementary figures and images for: Natural Transformation of Campylobacter jejuni Occurs Beyond Limits of Growth
Source: PLoS One. 2012 Sep 26;7(9):e45467. doi: 10.1371/journal.pone.0045467 (PMC3458841; doi:10.1371/journal.pone.0045467)

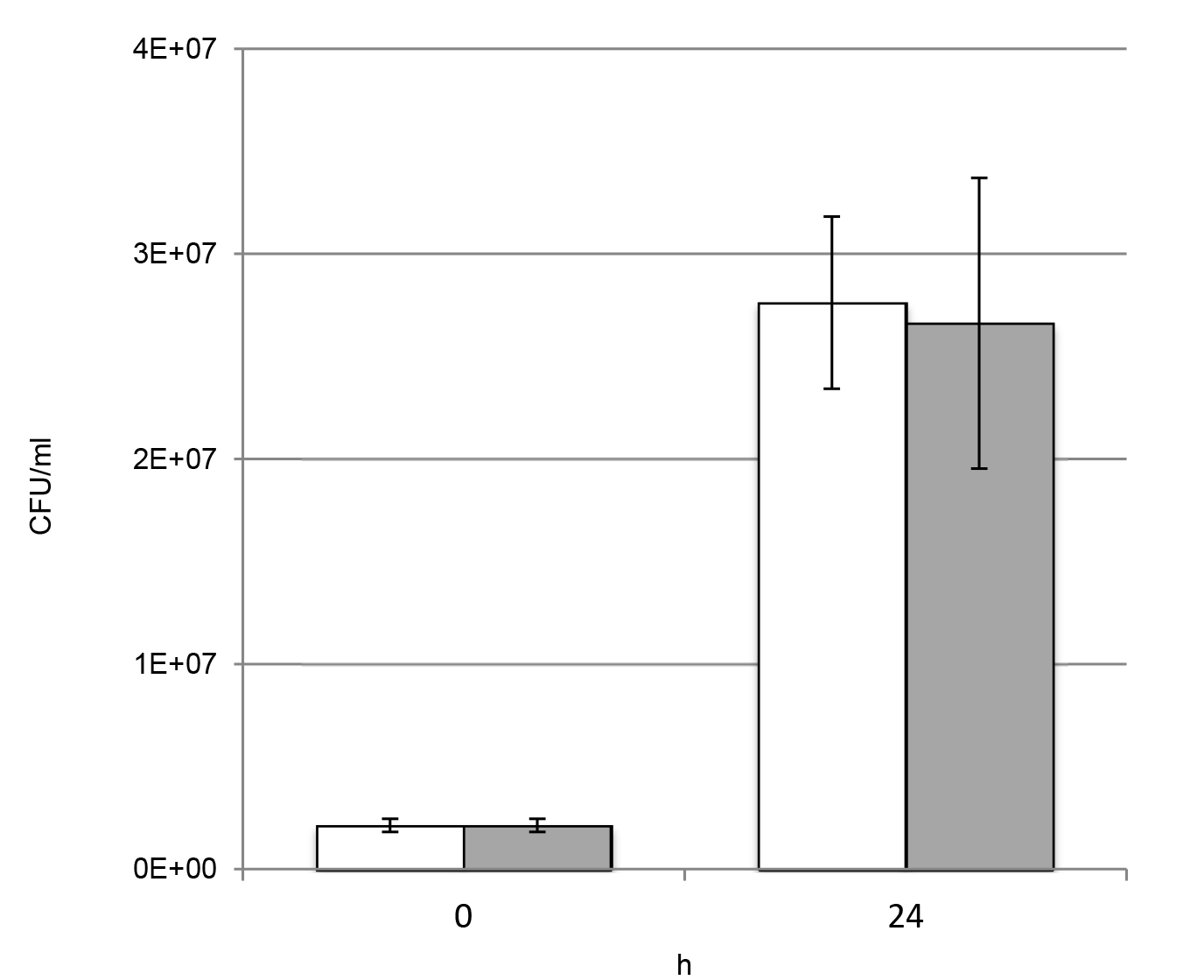

Supplement: Figure S1 — Growth of C. jejuni NCTC11168 is not supported by extracellular DNA. Growth of C. jejuni NCTC11168 in minimal media (MEMα medium without nucleotides catalog no 32561, Life Technologies) without (white) and with 10 µg/ml of isogenic chromosomal DNA (grey). The results represent a mean of four replicates with standard deviations. (TIF) [file pone.0045467.s001.tif]
