# Supplementary material for: Natural Transformation of Campylobacter jejuni Occurs Beyond Limits of Growth
Source: PLoS One. 2012 Sep 26;7(9):e45467. doi: 10.1371/journal.pone.0045467 (PMC3458841; doi:10.1371/journal.pone.0045467)
Supplement: Table S1 — DNA from A. butzleri and H. pylori cannot outcompete natural transformation of isogenic DNA. (PDF) [file pone.0045467.s002.pdf]

Table S1. DNA from *A. butzleri* and *H. pylori* cannot outcompete natural transformation of isogenic DNA.

C. jejuni Cam^R DNA[a]	*A. butzleri* DNA[b]	*H. pylori* DNA[c]	Cam^R Transformants/ml[d]
20 ng	-	-	$2.6x10^5 \pm 1.0x10^5$
20 ng	20 ng	-	$3.2x10^5 \pm 1.5x10^5$
20 ng	100 ng	-	$2.3x10^5 \pm 4.9x10^4$
20 ng	200 ng	-	$3.5x10^5 \pm 5.7x10^4$
20 ng	-	20 ng	$1.8x10^5 \pm 1.1x10^5$
20 ng	-	100 ng	$1.8x10^5 \pm 2.8x10^4$
20 ng	-	200 ng	$1.9x10^5 \pm 7.1x10^3$

[a] Chromosomal DNA from *C. jejuni* NCTC11168 *Δtlp1::Cam*^{R}

[b] Chromosomal DNA from *A. butzleri* ATCC4916

[c] Chromosomal DNA from *H. pylori* ATCC700392

[d] Results are mean of three replicates with standard deviation.
