# Supplementary material for: Natural Transformation of Campylobacter jejuni Occurs Beyond Limits of Growth
Source: PLoS One. 2012 Sep 26;7(9):e45467. doi: 10.1371/journal.pone.0045467 (PMC3458841; doi:10.1371/journal.pone.0045467)
Supplement: Table S2 — Natural transformation and survival of C. jejuni following DNA damage. (PDF) [file pone.0045467.s003.pdf]

Table S2. Natural transformation and survival of *C. jejuni* following DNA damage.

**DNA damaging agent**	**Concentration /intensity**	**Transformants/CFU**[a]	**Recovery incubation**[b]	**CFU/ml**
Negative control (MitC)	-	$2.1x10^{-6}$	-	$5.3x10^{8}$
Mitomycin C	10 ng/ml	$2.9x10^{-6}$	-	$4.9x10^{8}$
Mitomycin C	50 ng/ml	$1.5x10^{-6}$	-	$2.3x10^{8}$
Mitomycin C	100 ng/ml	$1.6x10^{-6}$	-	$1.9x10^{8}$
Mitomycin C	200 ng/ml	$3.1x10^{-6}$	-	$1.1x10^{8}$
Mitomycin C	500 ng/ml	$3.4x10^{-6}$	-	$5.9x10^{6}$
Negative control (UV)	0 J/m^2	-	2 h	$9.8x10^{8} \pm 3.5x10^{7}$
Negative control (UV)	0 J/m^2	$3.0x10^{-4} \pm 3.0x10^{-5}$	2 h + DNA	$9.5x10^{8} \pm 2.1x10^{8}$
UV	10 J/m^2	-	2 h	$2.3x10^{8} \pm 1.1x10^{7}$
UV	10 J/m^2	$3.6x10^{-4} \pm 3.9x10^{-5}$	2 h + DNA	$1.9x10^{8} \pm 2.5x10^{7}$
UV	15 J/m^2	-	2 h	$1.7x10^{8} \pm 3.2x10^{7}$
	15 J/m^2	$2.0x10^{-4} \pm 8.2x10^{-6}$	2 h + DNA	$1.6x10^{8} \pm 3.5x10^{6}$

UV	30 J/m^2	-	2 h	$4.6x10^7$ ± $5.3x10^6$
UV	30 J/m^2	$2.5x10^{-4}$± $5.2x10^{-5}$	2 h + DNA	$4.5x10^7$ ± $1.1x10^7$
UV	60 J/m^2	-	2 h	$4.3x10^6$ ± 0
UV	60 J/m^2	$1.4x10^{-4}$± $2.5x10^{-5}$	2 h + DNA	$4.5x10^6$ ± $1.8x10^6$
UV	80 J/m^2	-	2 h	$1.0x10^6$ ± $1.2x10^5$
UV	80 J/m^2	$1.4x10^{-4}$± $2.4x10^{-5}$	2 h + DNA	$7.8x10^5$ ± $7.1x10^4$

[a]Cam^R transformants per CFU.

[b]Following UV treatment, one volume of BHI and when indicated 2 μg/ml isogenic chromosomal DNA carrying a Cam^R marker was added to the culture. The bacteria were allowed to recover from the UV treatment for 2 h at 37° C in a microaerobic environment before scoring transformants and CFU/ml.

Results are representative from several independent experiments.
